# Reconciling the tension between the ‘global’ and the ‘local’ in medical education

**DOI:** 10.1111/medu.15740

**Published:** 2025-06-11

**Authors:** Birgit H. Fruhstorfer, Colin F. Macdougall

**Affiliations:** ^1^ Warwick Medical School University of Warwick Coventry UK

## Abstract

The authors highlight the need for all medical schools to effectively align the ‘global’ with the ‘local’ in curricula so students are prepared for practice in a globalised world.

Globalisation has clearly had an impact on how medical education is delivered. Medical schools now compete for applicants in a global space, and there is rapid flow of knowledge between distant institutions.^1^ In this issue of *Medical Education*, Edwards et al.^2^ offer a valuable contribution to the literature by reviewing the empirical evidence on the utility of transnational medical education programmes from the perspective of graduates from such programmes. In order to do so, the authors have broadened the definition of ‘transnational education’, which traditionally refers to the provision of education on a branch campus, by also including programmes with the primary goal to prepare students for practice in countries other than the country of study. Their work highlights the challenge of matching the curriculum content to the location of practice, which raises concerns about adequate preparedness for practice. More positively, findings also indicate that learning in an international context gives students the opportunity to develop intercultural skills, which are important competencies for professional practice anywhere in the world.

This study prompts us to revisit strong opposing forces that are at play in decisions on educational policy and curriculum design. In this commentary, we explore this tension from the perspectives of internationalisation and social accountability, which have been the subject of debate particularly over the past decade, with the aim to illuminate how this influences the understanding of what preparation for practice should entail.

The primary mandate of medical schools is to equip students with the competencies that enable them to provide safe and effective healthcare. Globalisation has had considerable implications for the work of health professionals who are now expected to address the health needs of diverse populations locally but who also have the opportunity to move across the world during their professional lives. As a result, Harden^1^ argued that insular models in which education considers medical practice only in relation to the local context are no longer appropriate to prepare students for medical practice in the 21st century. Instead, he argues that content of the curriculum should be ‘exemplified in the global context rather than that of a single country or locality’.^1^ The interconnectedness between distant communities means that there is a close relationship between local problems and global consequences, which needs considered to explain and find solutions for local health issues.^3^ Furthermore, graduates are expected to collaborate in multiprofessional teams to deliver healthcare to and across multicultural communities. Therefore, programmes also need to ensure that students develop attributes as a global citizen including global awareness and intercultural skills.^4^ The process of global citizen learning is facilitated when students are exposed to situations, which are out of their comfort zone and give the opportunity to experience interpersonal encounters with diverse others.^5^ When considering preparedness from this perspective, transnational programmes, as described by Edwards et al.,^2^ provide excellent opportunities to promote the development of these attributes, when students engage in learning in an unfamiliar context together with students from different countries.

However, at the same time, another force has emerged pulling in the opposite direction, evident in the seminal *Lancet* report on health professions education, when Frenk et al.^3^ note the challenge is ‘to adapt locally while harnessing the power of global flows of resources’. The concept of social accountability has gained increasing traction with calls for adopting ‘glocalisation’ practices, the adaptation of the global to local need, which involve a focus on local health needs for curriculum design.^6^ When looking at preparedness from this perspective, Edwards et al.'s study^2^ supports these principles, highlighting the mismatch between education and practice. However, an excessive focus on local social accountability has been criticised, with Gibbs and McLean^7^ endorsing the idea of global accountability to solve global issues effectively. Moreover, when exploring the tension between ‘global’ and ‘local’, Prideaux^8^ warns against considering social accountability as a remedy for solving health workforce issues.

insular models in which education considers medical practice only in relation to the local context are no longer appropriate

So, where are now? The pendulum seems to have swung more towards the ‘local’ rather than the ‘global’, which can be exemplified by recent UK Government proposals to implement a 4‐year undergraduate programme for school leavers in England as a quick fix to upscale the medical workforce.^9^ These plans are currently paused potentially due in part to concerns raised by several stakeholders in medical education. Considering the limited amount of time in a condensed programme, it becomes even more challenging to provide the education, which is necessary to produce a well‐rounded graduate.^10^ It is likely that study abroad opportunities, which prepare students for practice in a globalised world, would have been considered as expandable and removed from programmes. Furthermore, it has been questioned whether a shorter degree would be recognised internationally.^11^ Therefore, such efforts to prepare students rapidly for local practice seem at odds with aspirations of many students, who value mobility, and counterproductive to the aims of producing a graduate fit for practice in the 21st century.

The pendulum seems to have swung more towards the ‘local’ rather than the ‘global’

We should not escape the reality that we now live in a global space and we support the views of Edwards et al.^2^ that there is a need for educators ‘to move towards a more international outlook’. We note that the authors only included graduates from programmes, which train students deliberately for practice in other countries. However, many other medical schools accept international students, who intend to practise in their home country or another country following graduation in addition to local students, who of course may intend to practise elsewhere as well. These diverse groups of students will experience the same issues when transitioning to another country following graduation.

We argue that all medical schools need to ensure that their programmes prepare students for practice in a global space. This equally applies to usually the majority of students who plan to begin practice locally, as all students need to develop competencies that enable them to solve health issues in a complex world. As it is suggested by Edwards et al.,^2^ we advocate the need for including study abroad opportunities, such as international electives, in undergraduate programmes and for providing appropriate induction and orientation programmes in healthcare facilities to smooth the transition to local practice.

all medical schools need to ensure that their programmes prepare students for practice in a global space

We do not disagree with the idea that programmes should be designed to deliver content, which has a focus on the situation of healthcare in the local context, but this should occur without neglecting the global dimension of learning, which involves the consideration of health issues in a global context. However, this may not be enough if we want to tackle ubiquitous healthcare challenges, such as climate change, or mismatches between patient demands and resources. Training should go beyond the acquisition of technical skills with the aim to produce an ‘enlightened change agent’.^3^ To achieve this it is increasingly recognised that there is a need for integrating entrepreneurship education into the curriculum^12^, ^13^ to foster the development of important attributes, such as adaptation, creativity, problem‐solving and leadership, which are required to navigate challenges in a global world.

Edwards et al.'s study^2^ encourages us to revisit the tension between the ‘global’ and the ‘local’, which still requires to be resolved, so that we can move forward. As we continue to live in a globalised world, we need to find ways how we can effectively align the ‘global’ with the ‘local’ to produce graduates, who are able to find innovative solutions for complex issues in healthcare, wherever practice takes place. This needs to be considered for the curriculum design in all medical schools regardless of whether the orientation is more towards the training of local or international students.

the tension between the ‘global’ and the ‘local’, which still requires to be resolved, so that we can move forward

## AUTHOR CONTRIBUTIONS

**Birgit H. Fruhstorfer:** Conceptualisation; writing—original draft; writing—review and editing. **Colin F. Macdougall:** Conceptualisation; writing—review and editing.

## References

[1] Harden RM . International medical education and future directions: a global perspective. Acad Med. 2006;81(12 Suppl):S22‐S29. doi:10.1097/01.ACM.0000243411.19573.58 17086041

[2] Edwards G , Spooner M , Arnett R , Kelly H , Carr JCA , Illing J . Transnational medical education programmes and preparation for different country medical practice: a systematic review. Med Educ. 2025;59(9):924‐937. doi:10.1111/medu.15660 PMC12355643 40119725

[3] Frenk J , Chen L , Bhutta ZA , et al. Health professionals for a new century: transforming education to strengthen health systems in an interdependent world. Lancet. 2010;376(9756):1923‐1958. doi:10.1016/S0140-6736(10)61854-5 21112623

[4] Murdoch‐Eaton D , Redmond A , Bax N . Training healthcare professionals for the future: internationalism and effective inclusion of global health training. Med Teach. 2011;33(7):562‐569. 21696283 10.3109/0142159X.2011.578470

[5] Lilley K , Barker M , Harris N . Exploring the process of global citizen learning and the student mind‐set. J Stud Int Educ. 2015;19(3):225‐245. doi:10.1177/1028315314547822

[6] Ibrahim H , Abdel‐Razig S . Recalibrating our efforts: from globalisation to glocalisation of medical education. Postgrad Med J. 2021;97(1151):545‐546. doi:10.1136/postgradmedj-2020-139579 33541922

[7] Gibbs T , McLean M . Creating equal opportunities: the social accountability of medical education. Med Teach. 2011;33(8):620‐625. doi:10.3109/0142159X.2011.558537 21774647

[8] Prideaux D . The global‐local tension in medical education: turning 'think global, act local' on its head? Med Educ. 2019;53(1):25‐31. doi:10.1111/medu.13630 29974492

[9] Rimmer A . Four year medical degree set to launch in 2026, says NHS England. BMJ. 2024;385:q1312. doi:10.1136/bmj.q1312 38871382

[10] Lim JJ , Roberts C , Singh R , Wellington J . Bridging accelerated medical programmes and workforce demands: a critical evaluation of the four‐year direct entry medical degree. J R Soc Med. 2024;117(11):361‐365. doi:10.1177/01410768241289812 39451328 PMC11572710

[11] Finn GM , Brown MEL , Tiffin PA . Four year medical degrees in the UK. BMJ. 2024;386:q1630. doi:10.1136/bmj.q1630 39089848

[12] Ahmad H , Akram N . Should medical entrepreneurship be included in the curriculum? Br J Healthc Manag. 2018;24(1):648‐652.

[13] Mogaji IL , Raimi L . Embracing entrepreneurial innovation in medicine: the case for inclusion of entrepreneurship education in medical school curriculum. Ethics Med Public Health. 2025;33:101028.

